# Application of Biofire Filmarray Joint Infection Panel for Rapid Identification of Aetiology in a Necrotizing Fasciitis Case

**DOI:** 10.3390/diagnostics15010058

**Published:** 2024-12-29

**Authors:** Zoltán Tóth, Bence Balázs, Walter P. Pfliegler, Eszter Csoma, László Majoros, Dorka Szűcs, Renátó Kovács

**Affiliations:** 1 Medical Microbiology, Clinical Center, Faculty of Medicine, University of Debrecen, 4032 Debrecen, Hungary; 2 Department of Molecular Biotechnology and Microbiology, Faculty of Science and Technology, University of Debrecen, 4032 Debrecen, Hungary; 3 Department of Medical Microbiology, Faculty of Medicine, University of Debrecen, 4032 Debrecen, Hungary

**Keywords:** necrotising fasciitis, filmarray, hypervirulence, diagnostic, *Enterobacterales*

## Abstract

**Background**: Monomicrobial *Enterobacterales* necrotizing fasciitis is associated with exceedingly high mortality rates. Although effective antimicrobial therapy is an important part of treatment, the traditional microbiological diagnostic methods are not fast enough to meaningfully influence early therapeutic decisions. **Methods**: Here, we report the application of the BioMérieux Biofire Filmarray Joint Infection Panel (BFJIP) for the rapid detection of the causative agent and susceptibility prediction in such a case. Aside from the BFJIP-based rapid diagnostic approach and culturing, the whole genome sequencing (WGS) of the causative agent was performed using Illumina MiSeq and Oxford Nanopore MinION platforms. **Results**: The BFJIP indicated the presence of *K. pneumoniae*, without KPC, VIM, IMP, NDM, OXA-48 carbapenemase genes, and CTX-M-type extended-spectrum beta-lactamases. Based on the WGS data, the isolate belonged to the K1-capsule-type ST23, harboured a pNTUH-2044-like plasmid, and was positive for all the virulence factors associated with this lineage. The conventional susceptibility results were also in accordance with the BFJIP results; the isolate lacked any of these acquired resistance mechanisms. **Conclusions**: Despite this being the first case of the successful identification of pathogenic bacteria in necrotising fasciitis using this assay, the BFJIP may become a useful tool for rapid identification of pathogens in necrotising fasciitis cases and guiding antimicrobial therapy for better clinical outcomes.

## 1. Introduction

Necrotizing soft tissue infections (NSTI-s), including necrotising fasciitis (NF), are associated with high rates of mortality, generally ranging between 10 and 30% depending on the microbial aetiology and overall patient status/condition [1,2]. The therapeutic approach is surgical debridement with a concomitant administration of broad-spectrum antibiotics, mostly beta-lactams alone or in combination, covering possible causative organisms [3,4]. NF is historically classified into two groups: NF class I covers mixed aerobic/anaerobic infection, whereas NF class II covers beta-haemolytic streptococci infection with or without staphylococcal co-infection [2]. However, reports on uncommon causative agents (i.e., *Enterobacterales* species in monomicrobial infections) are increasingly prevalent, mostly due to ExPEC *Escherichia coli* and *Klebsiella pneumoniae* [5]. Kuehl et al. (2019) classify these monomicrobial Gram-negative infections as NF class III, together with those caused by *Vibrio* spp., and *Aeromonas* spp., and exceptionally high case fatality ratios ranging between 40 and 70% are reported for the former group [5,6,7]. While antibiotic treatment is not considered effective alone, early initiation of adequate antibiotics is associated with better clinical outcomes [1]. Nevertheless, the recent gold-standard microbiological diagnostic approach is plagued by long turnover [8], which may consequently result in inadequate antimicrobial treatment, while susceptibility results are pending in the case of resistant organisms. This may warrant application of novel rapid microbiological diagnostic tests for early presumptive identification of the causative agents and resistance determinants [2].

The BioMérieux Biofire Filmarray Joint Infection Panel (BFJIP) is a novel point-of-care multiplex PCR test, validated for identifying pathogens in septic arthritis, considered to be a difficult task, with a low number of viable microorganisms in the specimen and a broad range of potential causative agents [9]. Based on laboratory experiences, the assay has comparable sensitivity to classical microbiological methods in septic arthritis; moreover, it may significantly reduce the turnaround time (TAT) [9]. This diagnostic approach, however, may also identify several remarkable NSTI-related microorganisms, including *K. pneumoniae* and *Escherichia coli*; furthermore, it enables the detection of important beta-lactam resistance mechanisms of *Enterobacterales*, including CTX-M-type extended-spectrum beta-lactamases and the most common carbapenemases (IMP, VIM, OXA-48, NDM, and KPC) [10]. Moreover, the extensive range of targeted bacteria include but are not limited to *S. aureus* and *S. lugdunensis*, *Bacteroides fragilis*, *Clostridium perfringens*, Group A and B streptococci, and anaerobic Gram-positive cocci (*Peptoniphilus* spp., *Peptostreptococcus anaerobius*, and *Finegoldia magna*) [10]. Additionally, detection of mecA or mecC for methicillin resistant *S. aureus* and vancomycin resistance encoding genes vanA or vanB for enterococci is possible [10].

In this study, we report the successful use of the BFJIP for the timely detection of the causative agent and susceptibility prediction from a wound exudate obtained during the surgical debridement of a NF case.

## 2. Materials and Methods

A wound sample was sent to Medical Microbiology, University of Debrecen for immediate examination, obtained during surgical exploration and necrectomy of the gluteal region due to the suspicion of NF. The sample was shipped in a sterile container, and the sample could be described as a thick rust-coloured exudate, but it was transferable using Pasteur pipette and lacked visible tissue particles or signs of coagulation. Following a detailed consultation, the BFJIP assay was performed with 200 µL sample material according to the manufacturer’s instructions with concomitant Gram staining and culturing due to the rapidly deteriorating status of the patient. Standard culturing involved inoculation of the wound exudate onto Columbia agar supplemented with 5% sheep blood, chocolate agar, eosin methylene blue agar, Sabouraud-dextrose agar for aerobic culturing, and Schaedler agar supplemented with 5% horse blood for anaerobic culturing (CliniChem, Budapest, Hungary). Species-level identification of the isolate was performed by MALDI-TOF MS method (Matrix Assisted Laser desorption/Ionization Time OF Flight Mass Spectrometry) on a Bruker Biotyper (Bruker, Bremen, Germany) instrument in IVD mode of Biotyper Compass software. Susceptibility testing was performed according to EUCAST (European Committee on Antimicrobial Susceptibility Testing) disk diffusion methodology and interpreted accordingly [11,12]. Minimal inhibitory concentration (MIC) values for the tested agents were obtained using MIC Test Strips (Liofilchem, Roseto degli Abruzzi, Italy). Nucleic acid was isolated manually from overnight culture of the isolate with Qiagen DNeasy Blood & Tissue kit (Qiagen, Aarhus, Denmark) according to manufacturer’s instructions regarding Gram-negative microorganisms. DNA Library preparation was conducted using Illumina DNA Prep Kit (Illumina, San Diego, CA, USA) and Rapid Sequencing Kit V14 (Oxford Nanopore Technologies, Oxford, UK) for Illumina and Nanopore sequencing, respectively, from the same isolated nucleic acid. Illumina reads were filtered with fastp [13] (parameters: --cut_front 20, --cut_tail 20, --cut_window_size 5, --cut_mean_quality 15, -q 15, -u 50, -f 18, -w 8, -l 95) [13]. The genome was assembled de novo using a hybrid approach with Unicycler in conservative mode [14]. Sequence type, capsular serotype loci, virulence factors, resistance determinants, and plasmids in the assembled genome were identified by PathogenWatch [15]. Virulence factor DNA sequences of yersiniabactin, colibactin, salmochelin, aerobactin, and enterobactin were obtained from the VFDB [16] and visualized using ProkSee [17]. For comparative purposes, the assembled genome was compared to the genome of *K. pneumoniae* SGH10 as a reference [18], and *K. pneumoniae* NTUH-2044 [19]. The assembled genome is available under following accession number: CP136532-CP136533.

## 3. Results

**Figure 1:**
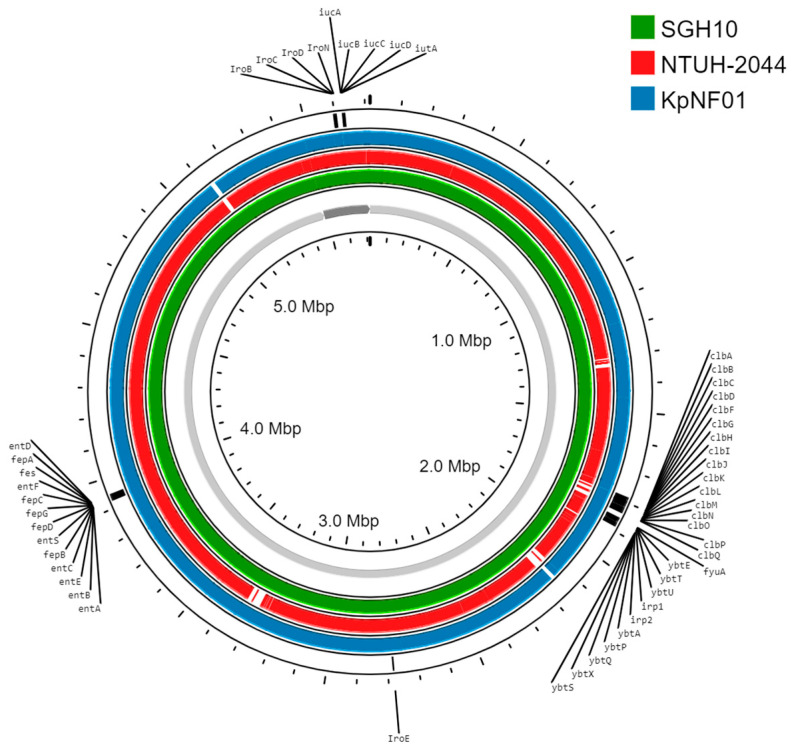
Proksee [17] comparison of the isolate (KpNF01) to *Klebsiella pneumoniae* SGH10 used as reference and *Klebsiella pneumoniae* NTUH-2044. Yersiniabactin, colibactin, salmochelin, aerobactin, and enterobactin gene clusters are also visualized.

The stained smear yielded high numbers of Gram-negative bacilli from the wound exudate, while the BFJIP indicated the presence of *K. pneumoniae* but did not detect KPC, VIM, IMP, NDM, OXA-48 carbapenemase genes, and CTX-M-type extended-spectrum beta-lactamases. The culture yielded *K. pneumoniae*, identified by the Bruker Biotyper as the sole microorganism, and the blood cultures obtained shortly after admission also yielded this microorganism a day after, with similar string test positivity and an identical antibiogram obtained using the EUCAST disk diffusion methodology. The susceptibility report confirmed the results of the BFJIP that the isolate was highly susceptible to third- and fourth-generation cephalosporins (cefotaxime, ceftazidime, and cefepime) and carbapenems (ertapenem, imipenem, and meropenem) with MIC values of ≤0.125 mg/L yet was resistant to ampicillin, to which *K. pneumoniae* is intrinsically resistant (Table 1). Whole genome sequencing further confirmed the results. According to the PathogenWatch database, the isolate belonged to the K1-capsule-type ST23 lineage, only harboured a pNTUH-2044-like plasmid, and all the virulence factors (yersiniabactin, aerobactin, salmochelin, colibactin, and enterobactin) were associated with this hypervirulent sequence type (Figure 1) [15,16,18] yet lacked beta-lactamases and other acquired resistance mechanisms except for the chromosomally encoded SHV-11 beta-lactamase responsible for aminopenicillin resistance.

## 4. Discussion

The obtained results highlight two interesting points: the possibility of rapid microbiological diagnosis of NSTIs by applying the BFJIP and the rare pathogen–clinical pathology pair, namely monomicrobial NF caused by HvKP, of which less than a hundred cases have been described so far and rarely observed in Europe [19].

Hypervirulent *K. pneumoniae* (HvKP) is a novel emerging pathotype that has shown a global expansion in recent decades; in addition, the acquisition of several resistance mechanisms has become a serious concern [18,20]. One highly prevalent lineage of HvKP is Clonal Complex 23, which contains ST23, ST26, ST57, and ST163 and is associated with several unusual clinical manifestations, e.g., liver abscess, endophthalmitis, meningitis, and NF, of which the former is the most frequently observed, in addition to diseases commonly caused by classical *K. pneumoniae* [21]. In contrast to the typical opportunistic pathogen classical *K. pneumoniae* isolates, HvKP strains may cause invasive diseases in otherwise healthy individuals in the community setting; although NF is a rare complication associated with several predisposing factors [19,21], HvKP NF is associated with a poor prognosis and a high mortality rate (~40–60%), especially among already hospitalized patients [5,19]. Based on epidemiological studies, diabetes mellitus, chronic liver disease, and corticosteroid therapy may predispose one to the development of HvKP NF [19]. In the case study, the patient had chronic liver disease as a predisposing factor, in line with the published literature [19]. The hypervirulence of these isolates is explained by their several siderophore systems for acquiring iron from the host, which is otherwise highly restricted [21]. The most common siderophore systems in HvKP strains are enterobactin, salmochelin, yersiniabactin, and aerobactin. ST23 strains typically encode the genotoxin colibactin, considered to be an important factor in translocation via biological barriers and found to be necessary to establish central nervous system infection in a murine model [22]. The K1 ST23 isolates were also found to be more virulent in a *Galleria mellonella* model compared to HvKP isolates of other sequence types [23]. According to the WGS data, the isolate in the present study belonged to the K1 capsule type ST23, harboured solely a pNTUH-2044-like plasmid, and was positive for all the virulence factors associated with this lineage (yersiniabactin, aerobactin, salmochelin, enterobactin, and colibactin). In the reported HvKP NF cases, the isolates mostly belonged to the K1 or K2 capsule types; nonetheless, other types had also been identified sporadically [4]. K1-capsule-type ST23 is highly prevalent in East Asia and the dominant lineage in the *K. pneumoniae*-associated NF cases of this geographical area [18,19,21]. In Europe, the reported cases where capsular serotypes were assessed were caused by K2 capsule types [24,25]. It is noteworthy that, to the best to our knowledge, this is the first published report of the K1 ST23 NF in Europe.

Acquisition of resistance mechanisms of this ST has rarely been observed since its description and has only been reported in the past ten years; however, since then, the emergence of carbapenemase-producer K1 ST23 shows a worrisome trend [18,26,27]. Additionally, the dissemination of novel hypervirulence plasmids among multi-drug-resistant *K. pneumonia strains* has also been reported, along with novel hybrid plasmids encoding both drug resistance and virulence gene clusters [28].

The gold-standard method for definitive microbiological diagnosis in the case of NSTI-s is Gram staining and culturing the specimen obtained during surgery via biopsy or blood cultures, which are plagued by long turnover [2,3,4]. Therefore, microbiological laboratory findings have little if any role in the initiated antibiotic therapy [2]. The slow TAT could be partially mitigated by using rapid molecular tests; however, there are no approved commercial point-of-care assays for rapid microbiological diagnosis of NF covering the broad range of potential aetiological microorganisms. Although rapid detection of bacterial species is possible, for example by PCR [1], experienced laboratory staff and equipment may be required on short notice. The recent guidelines on the empiric treatment of NF call for the administration of broad-spectrum antibiotics with aerobic and anaerobic coverage yet do not cover carbapenemase-producing isolates [3,4], which may lead to delayed initiation of effective antimicrobial therapy in such situations. Although NSTI-s caused by carbapenemase-producing *K. pneumoniae* are extremely rare so far, the global spread of strains resistant to carbapenems predicts an expected increase in cases [29]. Third-generation cephalosporin and piperacillin/tazobactam resistance, on the other hand, is rather common among *Enterobacterales* isolated from such infections [5,6,7]. Timely identification of the pathogens together with data on local resistance epidemiology and anamnestic information may prove to be helpful in tailoring antimicrobial therapy even without the prior detection of the exact resistance mechanism [2,7].

Compared to the routine microbiological workup in our case, the BJFIP results were available 10 h before the species-level identification by MALDI-TOF MS and 30 h before EUCAST disk diffusion susceptibility. The fulminant course of the disease is underlined by the fact that the patient had already died when the aerobic identification results became available despite the aggressive surgical debridement and seemingly adequate antimicrobial therapy. A lethal outcome even with adequate treatment is not common for HvKP NF, however [19]. Rapid identification of the pathogen may prove to be especially useful for these infections, which are often results of haematogenous spread from the primary foci, in contrast to those that are results of direct inoculation, e.g., in the case of *S. pyogenes* [19,21].

Despite the possible advantages regarding TAT reduction, this approach is not without limitations. As the assay’s intended use is not for microbiological workup of NSTI-s, somewhat understandably, several important causative microorganisms of such infections, e.g., *Aeromonas* and *Vibrio* species, the rarely encountered class IV NF-causing fungi (e.g., *Mucorales*), except for *Candida* spp., and several anaerobes reported from polymicrobial NSTI-s are not covered [2,11]. Considering the highly complex pathobiome observed in polymicrobial NSTI-s, however [2], it is questionable whether all the possible microorganisms can be identified concurrently in one such assay apart from next-generation sequencing methods. Indeed, performing WGS, which became an invaluable tool for epidemiological purposes in recent decades, on the obtained specimen is an appealing approach as, theoretically, all the possible pathogenic microorganisms could be identified together with the resistance determinants [30,31]. However, several obstacles must be overcome before WGS for rapid microbial diagnosis supersedes molecular point-of-care tests directly from the samples and becomes widely available. A few to mention are the expertise needed in high-quality nucleic acid isolation, library preparation, and quality control; furthermore, bioinformatic proficiency may be necessary as user-friendly platforms for such tasks are yet to be more available [31,32,33]. Considering all these factors, hands-on time and TAT as of now are still considerably longer, not to mention the required 0–24 equipment availability and price per sample of on-demand runs [32,33]. Nevertheless, as sequencers become more available and workflows become more simplified, WGS will likely become more commonly used in clinical microbiology laboratories [31]. Until then, for the BFJIP, simultaneous microscopical examination and anamnestic information, e.g., history of contact with natural waters, may also overcome the limited number of targets to some extent.

It should be noted that, although in the present case the assay successfully identified the causative organism from the wound exudate, the currently validated sample type for this assay is synovial fluid. The off-label use of the Biofire Filmarray in non-FDA-cleared clinical situations and sample types has been reported with promising results already [34,35]. Michos et al. reported the successful application of the Biofire Filmarray Blood Culture Identification Panel on synovial and pleural fluids, correctly identifying the causative agents, and Hirai et al. also reported positive results using the same panel applied to synovial fluids [30,31]. Furthermore, Benvenuto et al. recently described the application of the BFJIP on cerebrospinal fluid, abscess fluid, and tissue samples including skin and skin structure infections and the results are encouraging [36].

In our opinion, the TAT of about one and a half hours for this assay with a hands-on time of several minutes may significantly shorten the time needed to initiate adequate therapy in unusual NF cases caused by bacteria belonging to *Enterobacterales* and could become a useful complementary test in general microbial diagnostic workup of NSTI-s. However, extensive clinical validation is necessary with possible optimization of the protocol or until dedicated assays for NSTI-s are commercialized. With the emergence of HvKP worldwide and the observed convergence between virulence and resistance in *K. pneumoniae*, clinicians should be aware of the possibility of NSTI-s caused by isolates with antibiograms that are difficult to predict and not covered by recent empirical treatments.

## Figures and Tables

**Table 1:** Susceptibility testing results of the *Klebsiella pneumoniae* KpNF01 using EUCAST disk diffusion method and minimum inhibitory concentration (MIC) values using MIC Test Strips (MTSs).

Antibiotic	Interpretation by Disk Diffusion	MIC mg/L (MTS)
Ampicillin	Resistant	>64
Amoxicillin/Clavulanic acid	Susceptible	2
Piperacillin/Tazobactam	Susceptible	1
Cefotaxime	Susceptible	0.125
Ceftazidime	Susceptible	0.125
Cefepime	Susceptible	0.125
Meropenem	Susceptible	0.06
Imipenem	Susceptible	0.125
Ertapenem	Susceptible	0.06
Trimethoprim/Sulfamethoxazole	Susceptible	0.25
Ciprofloxacin	Susceptible	0.06
Gentamicin	Susceptible	1
Tobramycin	Susceptible	1
Amikacin	Susceptible	4
Ceftazidime/Avibactam	Susceptible	0.125
Ceftolozane/Tazobactam	Susceptible	0.125
Tigecycline ^1^	NA ^2^	0.25

^1^ No EUCAST breakpoint available. ^2^ NA: “not applicable”.

## Data Availability

Regarding the *K. pneumoniae* isolate tested, the Whole Genome Shotgun project has been deposited in DDBJ/ENA/GenBank under the Accession number of CP136532-CP136533.
